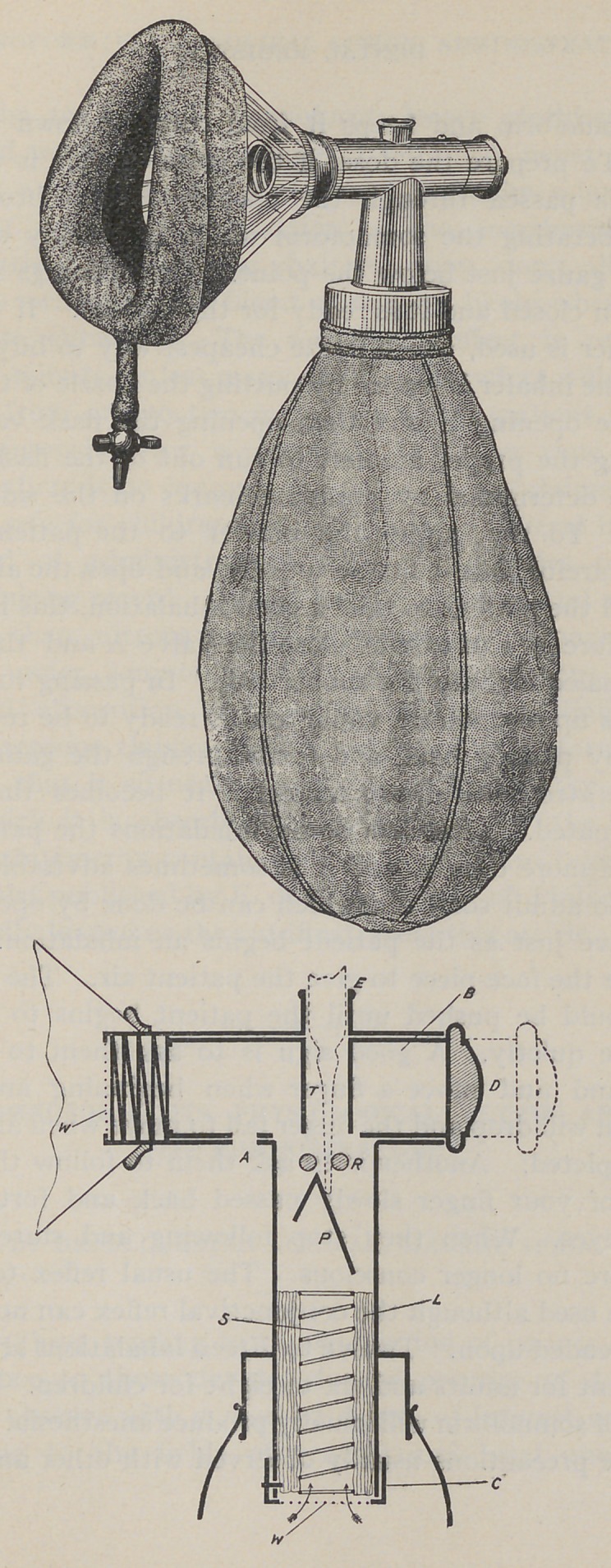# Somnoform and Its Administration

**Published:** 1905-01-15

**Authors:** 


### SOMNOFORM AND ITS ADMINISTRATION.

SOMNOFORM is a liquid, marketed in hermetically-sealed glass tubes, containing a little more than one dram, and in bottles with a self-closing valve, which contain about two ounces. The liquid volatilizes at room temperature very readily and the container must be well corked when not in use. It is made of

| | |
|---|---|
| Chloride of ethyl | 60% |
| Chloride of methyl | 35% |
| Bromide of ethyl | 5% |

It is administered with a special inhaler which we illustrate herewith. The inhaler consists of a celluloid cone, faced with flexible rubber to secure accurate adaptation to the face. The cone is attached to a metal angle or elbow tube which contains the valves and inhalant. To the lower end of the tube is attached a flexible rubber bag which furnishes all the air used in vaporizing the somnoform. The construction of the tube with location of the valves is shown in the second cut. On the bottom of the elbow is the air valve at A, which can be opened or closed by pressing in or out the inner tube with the thumb at D. At E is another opening, which is opened and closed at the same time and by the same means as the air valve A. By the opening E the somnoform is introduced in the chamber R and P which contains a piece of fine gauze which absorbs

E
B
D
T
W
A
R
P
S
L
C
W

the somnoform and keeps it from running down into the bag. To prepare the dose, if the glass capsule is used, its point is passed through the opening E and broken off, thus liberating the somnoform which is quickly absorbed by the gauze just below the point. The openings A and E are then closed and it is ready for the patient. If the flask container is used, which is the cheapest way to buy somnoform, the inhaler is loaded by putting the nozzle of the valve into the opening E and then opening the flask valve and allowing the proper amount to run out of the flask, which can be determined by graduate marks on the side of the bottle. To use, apply the inhaler to the patient's face, being careful that it fits accurately, and open the air vent A and tell the patient to take a good inhalation, this is mostly air; before he can exhale, close the valve A and this forces the exhaled air into the rubber bag. In passing to the bag it takes up somnoform vapor and is ready to be re-inhaled. Thus by passing back and forth through the gauze which is saturated with the somnoform it becomes thoroughly impregnated. After five or six inhalations the patient will breathe more deeply, and it is sometimes advisable at this point to admit some air, which can be done by opening the air valve just as the patient begins an inhalation. Never remove the face-piece to give the patient air. The anesthesia should be pushed until the patient begins to snore or breathe quietly. A good sign is to ask them to hold up the hand and move a finger when beginning anesthesia, the arm will drop and the finger fail to move when anesthesia is completed. Another is to ask them to follow the movement of your finger slowly passed back and forth before their eyes. When they stop following and stare fixedly, they are no longer conscious. The usual reflex tests may also be used although the conjunctival reflex can not always be depended upon. Twelve to fifteen inhalations are usually sufficient for adults and six to eight for children. One-half dram of somnoform will usually produce anesthesia.

The precautions usually observed with other anesthetics should be taken with somnoform; loosen clothing about neck and wrist, never give after a full meal, never give an overdose as it may create nausea and headache, a large dose does not increase the duration of the anesthesia, don't allow patient to leave the chair too soon, don't allow patients to swallow much blood as may be likely when many teeth are extracted. The anesthetic effect is produced in a half minute, or less many times. The effect will usually last for from one to two minutes and the patient is more or less insensible for a considerable longer time. It is claimed there is no cyanosis or stertor. It is claimed to be equally safe for children and old people. Several hundred thousand of administrations with no fatalities recorded is a promising record. It is better for children and aenemic persons than nitrous oxide. The pulse is stronger than normal under somnoform anesthesia. Its safety lies in its rapid absorption and almost as speedy elimination.

The reasons claimed for the safety of somnoform lie in the fact that it so nearly similates oxygen in the facility with which it is absorbed and eliminated by the tissues.

The statements contained in this article are taken from a pamphlet published by E. de Trey & Sons, of Philadelphia, who kindly loaned us the cut illustrating this article.

## Figures and Tables

**Figure f1:**